# Ice Age megafauna rock art in the Colombian Amazon?

**DOI:** 10.1098/rstb.2020.0496

**Published:** 2022-04-25

**Authors:** José Iriarte, Michael J. Ziegler, Alan K. Outram, Mark Robinson, Patrick Roberts, Francisco J. Aceituno, Gaspar Morcote-Ríos, T. Michael Keesey

**Affiliations:** ^1^ Department of Archaeology, University of Exeter, Exeter, UK; ^2^ Max Planck Institute for the Science of Human History, Jena, Germany; ^3^ Departamento de Antropología, Universidad de Antioquia, Medellín, Colombia; ^4^ Instituto de Ciencias Naturales, Universidad Nacional de Colombia, Bogotá, Colombia

**Keywords:** Pleistocene, rock art, megafauna, Amazonia, peopling of the Americas

## Abstract

Megafauna paintings have accompanied the earliest archaeological contexts across the continents, revealing a fundamental inter-relationship between early humans and megafauna during the global human expansion as unfamiliar landscapes were humanized and identities built into new territories. However, the identification of extinct megafauna from rock art is controversial. Here, we examine potential megafauna depictions in the rock art of Serranía de la Lindosa, Colombian Amazon, that includes a giant sloth, a gomphothere, a camelid, horses and three-toed ungulates with trunks. We argue that they are Ice Age rock art based on the (i) naturalistic appearance and diagnostic morphological features of the animal images, (ii) late Pleistocene archaeological dates from La Lindosa confirming the contemporaneity of humans and megafauna, (iii) recovery of ochre pigments in late Pleistocene archaeological strata, (iv) the presence of most megafauna identified in the region during the late Pleistocene as attested by archaeological and palaeontological records, and (v) widespread depiction of extinct megafauna in rock art across the Americas. Our findings contribute to the emerging picture of considerable geographical and stylistic variation of geometric and figurative rock art from early human occupations across South America. Lastly, we discuss the implications of our findings for understanding the early human history of tropical South America.

This article is part of the theme issue ‘Tropical forests in the deep human past’.

## 1. Introduction

Rock art has played a major role in Pleistocene archaeology, from discussions about the origins of behavioural ‘modernity' to the nature of human–animal relationships. Depictions of megafauna have accompanied the early human archaeological contexts across all of the continents (e.g. [1–3]), revealing a fundamental inter-relationship between early humans and megafauna during their global human expansion into new environments. However, the identification of extinct megafauna from rock art has been a source of considerable debate for archaeologists around the world [3–9]. Several potential Ice Age megafauna images have been suggested across the Americas [1,4,10–14], though the identification of the diagnostic physical features of the purported megafauna is controversial. The issue is further complicated as the majority of the paintings are not directly dated and, for the few that are, the ages are questioned or revised [13,15–17].

Here, we examine potential megafauna depictions in the rock art of Serranía de la Lindosa (hereafter La Lindosa), Guaviare Province, Colombia, which has recently produced a ‘media splash' [18] and generated some controversy regarding their validity and presentation. The authenticity of the potential Ice Age rock art has been called into question on two grounds. Trujillo Tellez *et al*. [19] argue that the paintings are so-well preserved that they cannot be that ancient. Meanwhile, Urbina & Peña [20] contend that some of the animals identified, in particular horses and camelids, are not Ice Age paintings, but are post-Columbian (after AD 1492), and that the giant sloth that we have identified represents a capybara (*Hydrochoerus hydrochaeris*).

In this paper, we address these criticisms, expand on the description of the megafauna from Morcote-Ríos *et al.* [9], and argue that there are sound grounds to consider these paintings as Ice Age megafauna. Our reasoning is based on: (i) the presence of a combination of salient physical features that are unique to the megafauna species being examined, (ii) the temporal overlap of humans and the megafauna species identified at La Lindosa based on the dates of human occupation of this region, (iii) the fact that the majority of the megafauna species being considered existed in northwest South America, as established by palaeontological and archaeological records, and (iv) the presence of ochre pigment fragments in the lower levels of our excavations at La Lindosa dating to 12.6 ka (calibrated years before the present using IntCal20 [21]). The paper begins with an introduction to the archaeology and rock art of the wider region. This is followed by a description of the La Lindosa rock art and the potential Ice Age animal depictions that resemble a giant sloth, a gomphothere, a camelid, horses and three-toe ungulates with trunks. This is accompanied by a brief review of the physical characteristics of the potential matching Ice Age megafauna candidates that roamed northwest South America and beyond at the end of the Ice Age from both palaeontological and archaeological contexts. Lastly, we discuss the implications of our findings for understanding the early human history of South America.

## 2. Early rock art in the Americas, study region and brief archaeological background

**Figure 1.**
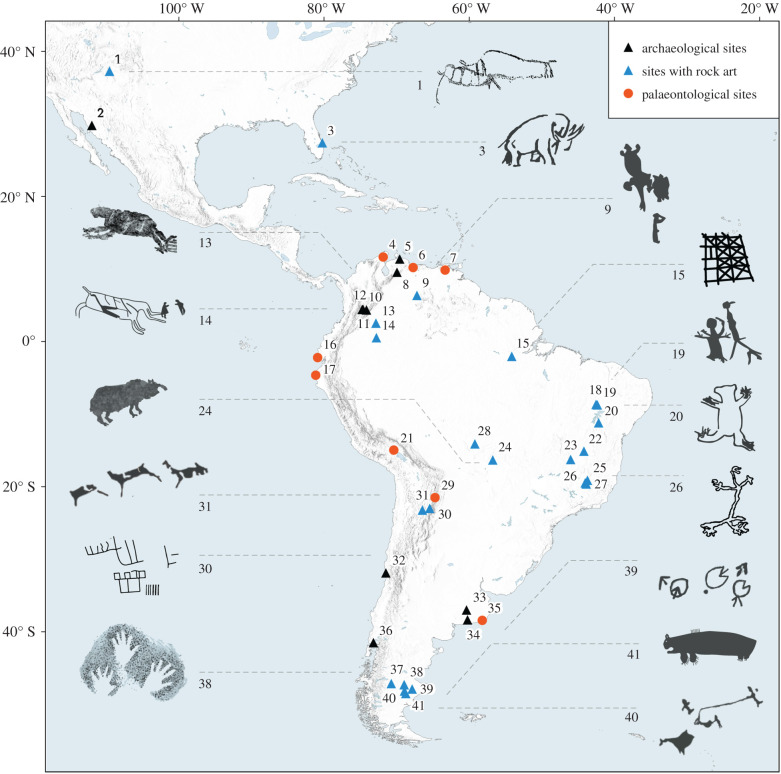
Map of the Americas showing most important LP-EH archaeological, rock and portable art, and palaeontological sites mentioned in the text where Ice Age megafauna has been documented or portrayed: 1. Upper Sand Island site; 2. Fin del Mundo; 3. Vero Beach; 4. Ware Formation; 5. Taima-Taima; 6. Lake Valencia; 7. El Breal de Orocual; 8. El Vano; 9. Cerro Gavilán; 10. Totumo; 11. Pubenza; 12. Tibitó; 13. Serranía la Lindosa; 14. Chiribiquete; 15. Caverna da Pedra Pintada; 16. Tanque Loma; 17. Talara; 18. Serra da Capivara; 19. Toca da Bastiana; 20. Bahia; 21. Casa del Diablo; 22. Lapa do Boquete; 23. Lapa do Gentio; 24. Santa Elina; 25. Santana do Riacho; 26. Lapa do Santo; 27. Lagoa Santa region; 28. Abrigo do Sol; 29. Tarija; 30. Inca Cueva; 31. Hornillos 2; 32. Los Vilos; 33. Campo Laborde; 34. Arroyo Seco 2; 35. Centinela del Mar; 36. Monte Verde; 37. Cueva de las Manos; 38. Los Toldos; 39. Piedra Museo; 40. Cerro Tres Tetas; 41. El Ceibo. (Online version in colour.)

A diversity of late Pleistocene/early Holocene (LP-EH) archaeological sites across South America occur in rock shelters associated with rock paintings that include naturalistic images of animals, geometric designs and hand negatives (figure 1; see summary in the electronic supplementary material). Our study site, the Serranía de la Lindosa is a 20 km^2^ rocky outcrop located in the Department of Guaviare, in the northwest of the Colombian Amazon. It is situated along the banks of the Guayabero/Guaviare River in today's transitional ecotone between the savannahs of the Orinoco and the Amazon rainforest. The first reports of rock art date back to the 1940s when the French explorer Alain Gheerbant [22] recorded them during an expedition in search of the source of the Orinoco River. Subsequent studies have been carried out at Cerro Azul, Nuevo Tolima and Raudal del Guayabero since the 1980s, which has given greater archaeological visibility to the rock art and attracted the attention of national and international researchers alike [20,23,24]. The first excavations in La Lindosa were undertaken by Correal [25] at the Angosturas II painted rock shelter at Raudal del Guayabero, which was radiocarbon dated between approximately 8.1–4.0 ka. During three archaeological field seasons (2015, 2017 and 2018), we carried out excavations in three rock shelters (Cerro Azul, Cerro Montoya and Limoncillos), where lithic artefacts, charred seeds, animal remains, ochre fragments and charcoal were recovered in the stratigraphic deposits of these sites [9]. These three rock shelters exhibit large concentrations of rock art paintings, though at varying degrees of preservation. The dating of these sites allowed us to establish the chronological framework for the rock art of La Lindosa, with dates ranging from the late Pleistocene approximately 12.6 ka to the European arrival approximately 1478*–*1642 AD [9]. The lithic technology of the earliest archaeological strata consists of an expedient unifacial technology mainly composed of small (smaller than 7 cm long) used and retouched flakes made on local chert and quartz. Faunal and plant remains point to a generalized economy exploiting both aquatic and terrestrial environments, dominated by fishes, small mammals and reptiles, as well as a diversity of palms and tree fruits [9]. During our recent survey of the region, we also discovered a whole new section of the western area of La Lindosa with five new panels along approximately 1 km of rock walls oriented northwest-southeast, facing southwest and standing approximately 370–470 m above sea level.

## 3. The La Lindosa rock art

**Figure 2.**
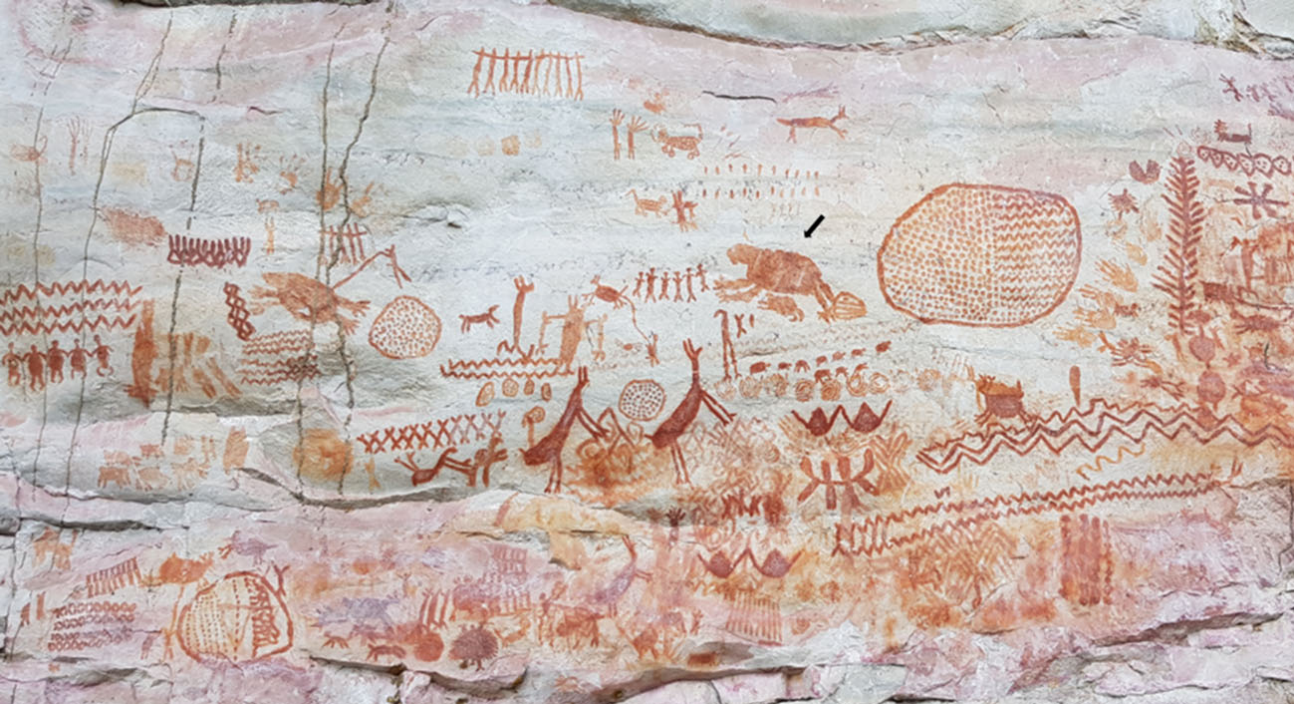
Las Dantas panel at Cerro Azul, La Lindosa (arrow points to proposed giant sloth painting). (Online version in colour.)

The La Lindosa rocky outcrop contains thousands of paintings which, along with the ones reported for Chiribiquete National Park, represent one of the richest rock art regions in the Americas (figure 1) [10,20,23,24,26]. Unlike the Upper Palaeolithic artists of Europe who chose to paint in deep dark caves, these early Amazonians painted in open rock shelters. Preservation of the paintings is highly variable, with images extremely faded or lost where exposed to the elements, whereas panels protected from prevailing wind and rain retain their vibrancy. The vertical rock walls reach up to 10 metres high. Some contain recessed or ‘hidden' panels that cannot be seen from ground level. Paintings on these panels are only visible today to an experienced climber with appropriate gear or with the advantage of drone technology. Painted images of what appear to be ladders or scaffolding perhaps provide clues as to how the early artists used natural resources to reach these locations. Ochre pigments provide the characteristic reddish-terracotta colour of the paintings. The most abundant motifs depicted in La Lindosa are zoomorphic, geometric, anthropomorphic and vegetal themes. Many of the images depict hunting and ritual scenes, showing humans interacting with plants, and forest and savannah animals. The animal paintings consist of naturalistic outlines and/or infilled designs (figure 2). It is apparent that La Lindosa artists recognized the importance of certain details of physiology and behaviour for signalling specific taxa for the audience. The realism of the depictions allows for taxonomic identification of a diversity of zoological groups, including fishes, rays, turtles, caimans, capybaras, deer, porcupines, felines, possible canids, monkeys and birds. Among this rich pictorial variety of animals, there are some intriguing images that appear to represent extinct megafauna including a giant ground sloth, gomphothere, camelids, horses and three-toed ungulates with trunks that bear some resemblance to some extinct megafauna such as *Xenorhinotherium* or *Macrauchenia*.

## 4. The megafauna paintings

Below we describe these potential Ice Age megafauna paintings in detail and provide a summary account of the palaeontological and archaeological contexts for these animal taxa that supports their identification in the panels.

### (a) Giant ground sloth (Megatheriidae)

**Figure 3.**
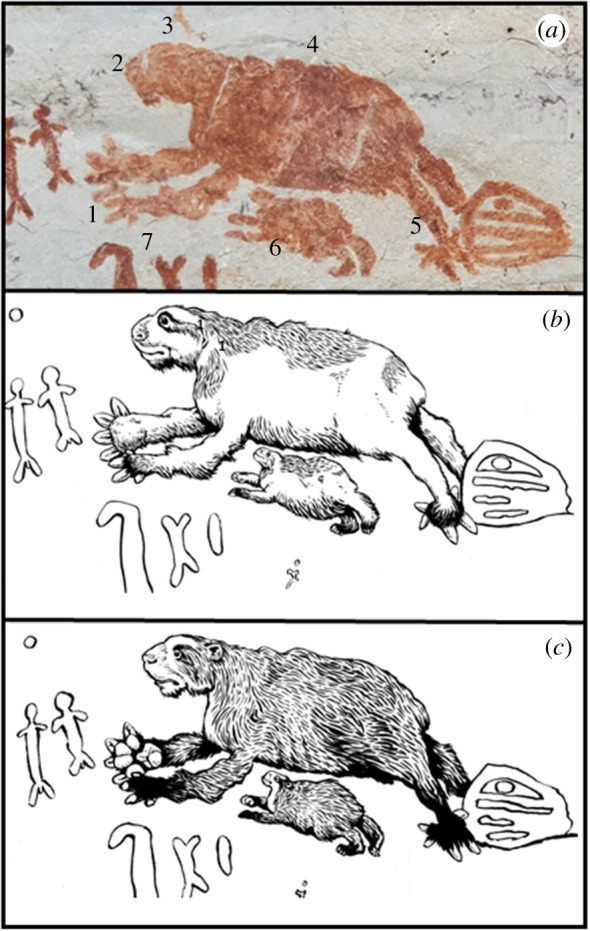
(*a*) Giant sloth painting at La Lindosa: 1. massive claws; 2. short rostrum; 3. large head; 4. robust thorax; 5. inverted pes; 6. offspring; 7. miniature men. (*b*) Artistic reconstruction of *Eremotherium* patterned after its closest living relative *Bradypus*. (*c*) Artistic reconstruction of *Arctotherium* patterned after its closest living relative, *Tremarctos ornatus* (Mike Keesey). Keesey's reconstructions are figurative works of art, where he took the liberty of adding features that are not visible in the rock art like fur, ear canals and wrinkles, among other features. (Online version in colour.)

This painting is located in Cerro Azul on the middle section of the panel locally known as ‘Panel de las Dantas’ (Panel of the Tapirs) (figure 2), as the wall is dominated by two almost life-size supposed tapirs facing each other. This panel includes the intriguing animal depicted in figure 3. Its overall morphology, large head, short rostrum, robust thorax, reduced number of digits on the pes and prominent claws recall a giant ground sloth. Presented in a quadrupedal stance, the sizable forearms appear to be longer than the hindlimbs. The manus consists of three to four digits extended distally, whereas the pes appears to have five digits with varied orientation. Notably, the depicted animal appears to exhibit pedolaterality, that is, the characteristic inverted pes, where the dorsal surface of the foot faces laterally and the planar surface of the foot faces medially. Three transversal lines compartmentalize the body in four parts and give the figure an appearance of surficial texture. The white mark on its head seems to be representing an eye. Behind the head, there appears to be a few protuberances along the dorsal surface that might represent prominent scapula and shoulder musculature. The animal is accompanied by an offspring and surrounded by animated miniature men, some of whom extend their arms towards the painting. The relationship of the animal with the men appears to be central to the artist's message. The comparatively smaller illustrated humans that accompany the animal appear to provide a perspective on scale that points to the sheer size of the specimen.

Although Urbina & Peña [20] believe the image represents a capybara (*Hydrochoerus hydrochaeris*), there are significant morphological features characteristic of an extinct giant ground sloth that are depicted in the painting. The general morphology and apparent size of the painted animal also bears some resemblance to extinct ursid taxa (i.e. *Arctotherium wingei*), which are known from LP-EH localities in Central [27] and northwestern South America [28]. Of note, the extant ursid (*Tremarctos ornatus*) has been documented from Holocene sites in Colombia dated to approximately 4.0–2.7 ka [28]. Therefore, we have included an artistic representation in figure 3*c* for comparison. However, despite the apparent lack of a pronounced tail, the elongated forearms and a reduced number of digits on the manus in the painting are anatomical features generally dissimilar to the extinct South American *Arctotherium* and more consistent with giant ground sloth taxa (see the electronic supplementary material for details of taxonomy, detailed morphological characteristics and habit).

Collectively, the Pan-American giant ground sloths *Eremotherium*, *Megatherium* and *Glossotherium* had a pervasive geographical distribution and are among the most common megafauna taxa found in Pleistocene to early Holocene sites throughout tropical and equatorial regions of South America [29,30]. There is a temporal overlap between records of early humans arriving in South America and the extinct giant ground sloth megafauna [31] showing a complex relationship including the hunting (Campo Laborde: [32]) and scavenging [33] of these large megaherbivores. Within northwest South America, *Glossotherium* fossils were recovered from the Taima-Taima site, Venezuela, which was most likely situated in a semi-arid, xerophytic ‘brush' habitat, and dated between approximately 15.2*–*17.3 ka [34,35]. Near Taima-Taima, fossils of *Eremotherium rusconi* were found at the El Vano locality directly dated to approximately 11.8 ka and associated with El Jobo artefacts [36]. In Colombia, the fossil record of the Pubenza locality, dated between approximately 19.9 and 16.2 ka, includes fragmentary elements of *Megatherium* as well as archeological obsidian elements that suggest the presence of human activity [37]. Ground sloth fossils from adults and juveniles associated with lithic artefacts have been excavated at the Totumo site and are correlated with a direct date on a gomphothere (*Notiomastodon* sp.?) bone that yielded a date of approximately 7.0 ka [38]. Further dating of this bone assemblage need to be carried out to confirm this anomalous late date for megafaunal survival into the Holocene. Three osteoderms of the giant ground sloth *Glossotherium lettsomi* flattened by abrasion and perforated like pendants were recovered from the Santa Elina site in levels dating to 27 ka [39]. Although relatively little is known about behaviour or social structure of extinct giant ground sloths, new evidence from a monotypic late Pleistocene locality, Tanque Loma, coastal southwest Ecuador, provides insights into the potential gregarious nature of these megafauna [40]. Researchers interpret the Tanque Loma site as a catastrophic fossil assemblage composed of adult and juvenile *Eremotherium laurillardi* individuals and suggest that they may have gathered in intergenerational groups. Indirectly, this gives support to the interpretation of the giant sloth image with the juvenile offspring.

### (b) Proboscidean (Gomphotheriidae)

**Figure 4.**
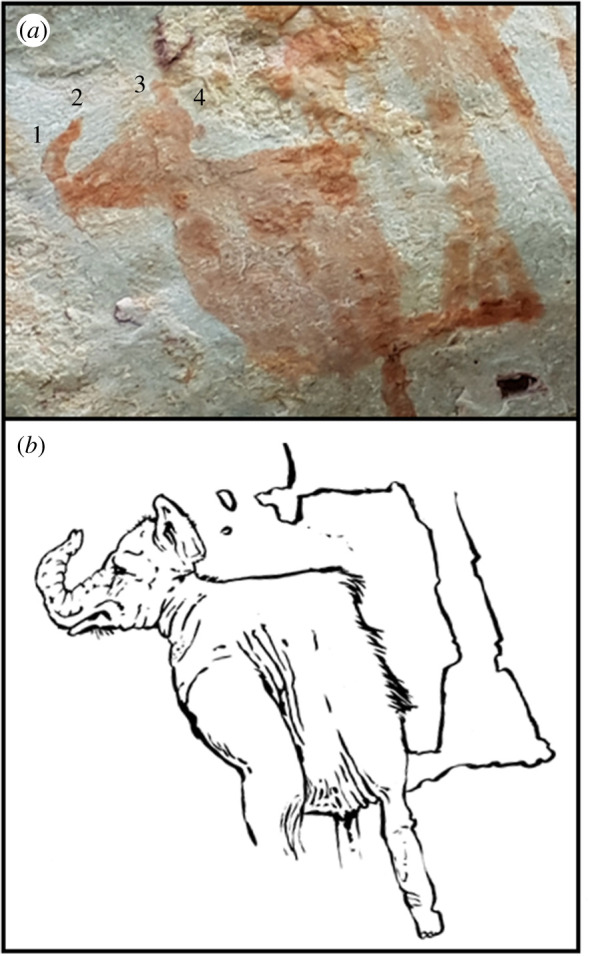
(*a*) Gomphothere painting at La Lindosa: 1. proboscis; 2. fingers; 3. flared ears?; 4. moderately domed head. (*b*) Artistic reconstruction (Mike Keesey). (Online version in colour.)

Located in the newly discovered section of painted walls on the western flanks of La Lindosa, our team found an animal rendering that is suggestive of a proboscidean (figure 4). Displayed in lateral view, the silhouetted image is composed of a relatively detailed head and robust posterior. Overall, the head is shorter dorsoventrally than anteroposteriorly. The head exhibits a rounded apex and is accented by a semi-curved protuberance that may denote flared ears. A muscular proboscis tapers to its distal terminus that appears bifurcated and could represent the ‘fingers’ at the tip of the prehensile trunk. While the specimen admittedly does not have discernable postcranial elements or the presence of characteristic tusks, it displays other anatomical features representative of South American gomphotheres, such as a distinct proboscis, a jaw that is not distinctively downturned, and a moderately domed head (see the electronic supplementary material for details of taxonomy, detailed morphological characteristics and habit). In all fairness, it is worth noting that certain details of the painting such as the dent between the apex of the head, the tips of the ears, and the trunk could easily be faint or damaged pigment. Future examination with image processing algorithms for rock art research and detailed superposition analysis could work out these issues. Regarding the flared ears, it also should be noted that the placement of auricles on Proboscidea skulls is always much lower than on the potential mastodon painting.

Other possible proboscideans have been identified in several rock and portable art media in the Americas. In the nearby Chiribiquete National Park, a zoomorphic figure with clear tusks has been tentatively identified as an ‘elephant' [10]. Petroglyphs of proboscideans have been recorded in the Upper Sand island site in Utah [1] and an engraved image of a proboscidean was engraved on a fragmented fossil bone collected at Vero beach in Florida [41] (but see [17] for a critical review) (figure 1).

In South America, *Notiomastodon platensis* had a more widespread distribution than *Cuvieronius hyodon*, which was largely restricted to the Andean region, but with similarly mixed diets (i.e. woodland, mixed, grassland) [42–44]. Despite overlapping geographical distributions in the mid and northwestern regions of South America, these South American gomphotheres have rarely been recorded in the same locality [45,46]. *Notiomastodon platensis* has a continuous record throughout the early Pleistocene until its extinction in the early Holocene, which is probably attributed in part to early humans in South America. Mothé *et al.* [47] report evidence of megafaunal killing by humans represented by a perforator tool embedded in the skull of a *N. platensis* calf from Lagoa Santa Karst locality in Brazil. *Cuvieronius* is also present in the Monte Verde site now dated to approximately 14.6 ka [48,49].

Currently, the earliest potential evidence of interactions between humans and gomphotheres in northwest South America comes from the Pubenza site of the Middle Magdalena River. Here unifacial tools co-occurred with *Haplomastodon waringi* (= *Notiomastodon*) remains in strata dated to approximately 19.9 and 16.2 ka [50]. In close proximity to Pubenza, at the Totumo site, Correal [51] unearthed a circular feature containing *Haplomastodon* (= *Notiomastodon*) in association with lithic artefacts. A direct date on the gomphothere bone yielded a date of approximately 6.9 ka. More work and dating of these sites need to be carried out to validate and confirm the cultural association of these contexts. At the Tibitó site in the Bogotá plateau, Correal [52] excavated three archaeological features in which burnt and unburnt bones of proboscideans (*Cuvieronius* and *Haplomastodon*) were mixed with stone artefacts. A radiocarbon assay on a single bone yielded a date of approximately 13.6 ka. At Taima-Taima, the mid-section of an El Jobo projectile point was excavated within the obturator foramen of a juvenile *Haplomastodon* (= *Notiomastodon*) in context dating to approximately 15.2*–*17.3 ka [34]. To the north, at the Fin del Mundo site in Sonora, Mexico, remains of a *Cuvieronius* sp. gomphothere have been dated to approximately 13.4 ka in association with Clovis material culture [53].

### (c) Horse (Equidae)

**Figure 5.**
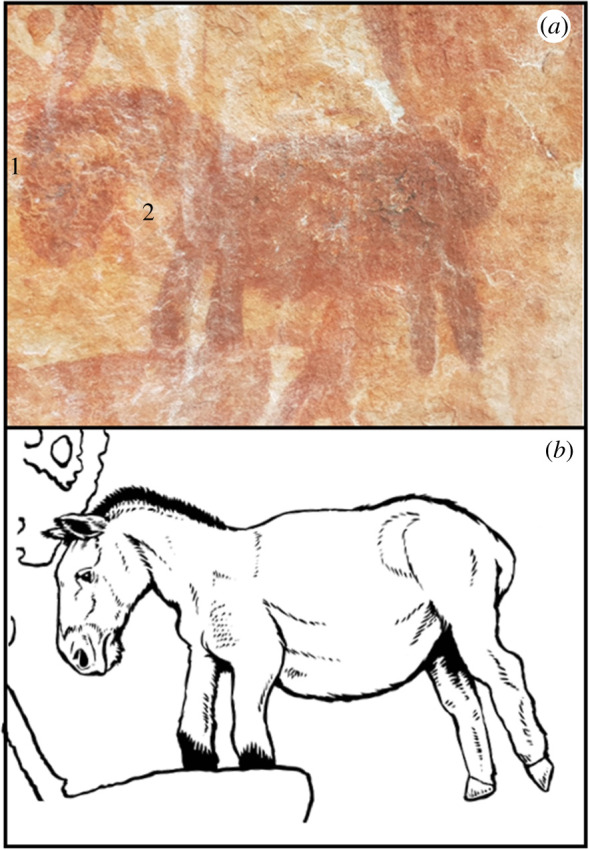
(*a*) Horse painting at La Lindosa: 1. large head; 2. robust neck. (*b*) Artistic reconstruction of *Hippidion* patterned after wild *Equus* (Mike Keesey). (Online version in colour.)

Potential representations of horses were painted on panel 1 and panel 3 of Cerro Azul (figure 5) (see also [20]), on the walls adjacent to our excavations. The horse from panel 1 is located on a high recessed panel, 4 m above the ground surface. The horses exhibit a large, heavy head and robust neck characteristic of American Ice Age horses (see the electronic supplementary material for details of taxonomy, detailed morphological characteristics and habit). They are slightly convex in the facial region running from frontal to the nose, while in domestic *Equus caballus* this is far less common, depending upon breed, with both the neck and head being considerably more slender. A slender, dished face is associated with Arabian/Persian horse breeds, and ancient genomics demonstrates increased Arabian/Persian ancestry in Europe from the early medieval period, becoming common by the period of European colonization of the Americas [54]. The horses in both panels 1 and 3 appear to have stiff manes and, while such brush-like manes are not an essential characteristic of an Ice Age horse, such depictions predominate, in sharp contrast to domestic *Eq. caballus*. Overall, the head is relatively large in comparison with the body and the limbs are stocky. In the painting, there are no discernible digits on the distal most portion of the limbs which would be supportive of monodactyl equids. However, the identification of Ice Age horses is not straightforward because horses became extinct at the end of the Pleistocene and were later reintroduced by Europeans to the Americas. Unlike Urbina & Peña [20], who interpret them as European horses, we tend to favour the hypothesis that these are Pleistocene horses. Our judgement is based both on the anatomical features of the horses, as noted above, but also on the observation that the majority of indigenous post-Columbian pictographs of domestic Old World *Eq. caballus* horses are painted with human riders. The human riders was the aspect that most caught the attention and curiosity of late Holocene Native Americans when they saw horses for the first time [13,55]. In addition, engravings that resemble hoof prints have been recorded at the LP-EH Piedra Museo site [56] (figure 1).

All the South American representatives of the genera *Equus* and *Hippidion* became extinct at the LP-EH transition when humans were already present in most of the environments on the continent [57] and are well documented in archeological contexts, especially in southern South America [58,59]. At the Piedra Museo site in Argentina, a *Hippidion* sp*.* humerus dated to approximately 11.6 ka displayed cut marks and indicates that equids may have been a more important resource for early humans in South America than previously thought [60]. Moreover, there is evidence of human hunting/scavenging of *Equus* material recorded from the Arroyo Seco 2 locality in the Pampas region dated to approximately 14.0 ka [61]. In the central Andes, direct dating of *Hippidion* bones at the Casa del Diablo cave in the Peruvian puna reported a date of approximately 15.3 ka and further illustrates their likely coexistence with early humans in the region [62]. Furthermore, *Equus* sp. was documented in the Venezuelan semiarid xerophitic shrub at the Taima-Taima locality in contexts dating from approximately 17.3*–*15.2 ka [34,35]. At the Tibitó locality, *Equus* (*Amerhippus*) *lasallei* bones were found physically associated with lithic artefacts and were dated to approximately 13.6 ka [58]. There are other fossil occurrences of *Equus* known from localities in northwestern South America (e.g. Colombia, Ecuador, Peru, Venezuela; see [63]), but many lack a refined chronology.

### (d) Camelid (Camelidae)

**Figure 6.**
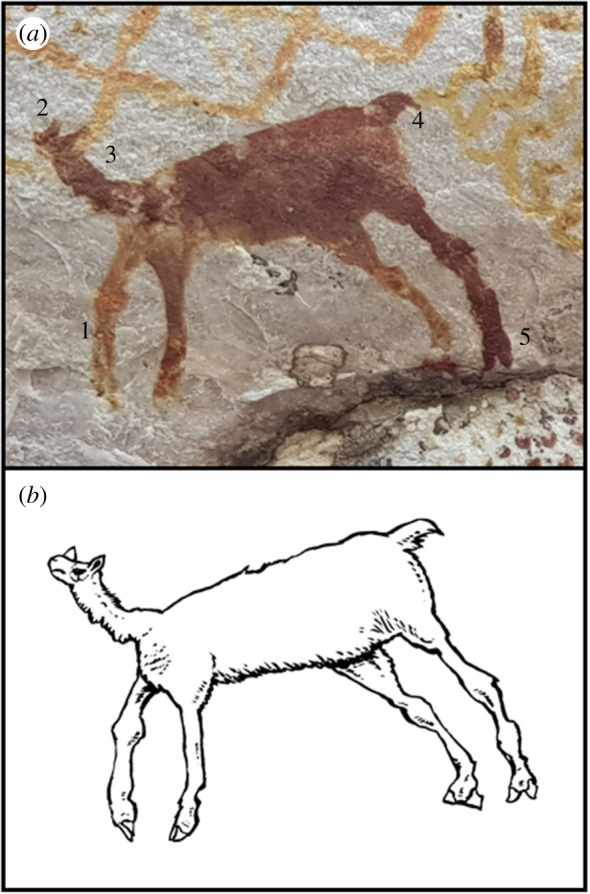
(*a*) Camelid painting at La Lindosa: 1. front legs smaller than rear legs; 2. small head; 3. long thin neck; 4. ‘camelid' tail; 5. two toes. (*b*) Artistic reconstruction of *Palaeolama* (Mike Keesey). (Online version in colour.)

Located in the Nuevo Tolima panel at La Lindosa, this image (figure 6*a*) captures a quadrupedal animal with morphological elements characteristic of camelid taxa. A distinctively small head is adorned by two moderately tapered protrusions that probably represent ears. The elongated neck appears to have uniform width and extends into the posterior of the animal which is composed of more diagnostic postcranial features. The animal displays a short tail and semi-robust extremities. Specifically, the forelimbs are shorter than the hindlimbs which is a characteristic attribute of non-domesticated camelids previously documented in formative South American rock art [64]. Moreover, the detailed limbs appear to be segmented with elongated upper and robust lower sections demarcated by a rounded and posteriorly extended protuberance. This segmentation may represent pronounced carpus and tarsus on the forelimbs and hindlimbs, respectively. Although preservation and orientation of the painting limits observation, the distal most portion of a hindlimb clearly illustrates a foot composed of two digits which is typical of the even-toed ungulates Artiodactyla. Other illustrations of camelids are common from early sites in Patagonia [13]. Overall, the depicted animal is markedly distinct to all the cervids (deer) that are profusely painted at the La Lindosa site and exhibits characteristics of South American camelids, possibly representing *Palaeolama* (see the electronic supplementary material for details of taxonomy, detailed morphological characteristics and habit). It should be noted, however, that *Palaeolama* itself is noted for its elongated and robust cranium, which does not closely match the painting. In addition, a point to keep in mind is that both *Palaeolama* and *Hemiauchenia* have somewhat similar cranial and dental morphologies, and their distinctions would be lost in simple artistic renditions as the one in the painting. Last but not least, the fact that the artistic rendering could represent *Lama* or *Vicugna* should not be discarded. As of yet, no excavations have been conducted in the Nuevo Tolima site, and as a result, we do not have any chronological information about this locality.

There is little evidence in the area for the coexistence of humans and camelids. In the Pampas region, *Hemiauchenia* bones were located at a site with lithic artefacts and megafauna specimens dated to approximately 14*–*11.2 ka, but without any evidence to support behavioural association with humans [61]. At the Los Vilos site in southern Chile, *Palaeolama* sp. bones co-occurred with questionable association of lithic artefacts in strata dated to approximately 12.0 ka which would support a temporal overlap between early human settlers and Lamini on the South American landscape [65]. In the northwestern region of South America, there is no association of *Palaeolama* bones with humans, but *Palaeolama* bones have been found in the fossiliferous tar deposits from the Pleistocene El Breal de Orocual locality of Venezuela [66]. Moreover, the Talara site in the Peruvian Pacific coast supports the presence of *Palaeolama* among other Pleistocene megafauna that have been dated to approximately 17.6*–*16.5 ka [67,68]. It must be mentioned that extant Lamini taxa further complicate matters because the fossil forms of *Lama* are reported to have their origin in the Andean region [69] and became domesticated in the Holocene (approx. 5*–*3.8 ka) [70,71]. Similarly, *Vicugna* were probably first domesticated in highland Peru during the early to mid-Holocene (approx. 7.0*–*6.9 ka) [71,72].

### (e) Macraucheniid (Macraucheniidae)

**Figure 7.**
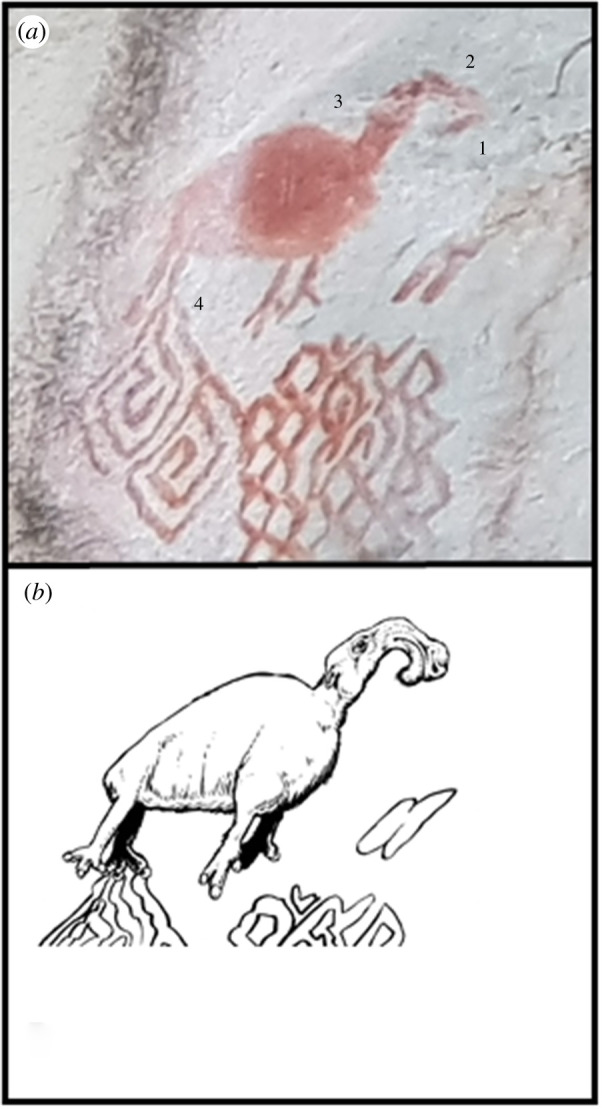
(*a*) Macraucheniid paintings at La Lindosa: 1. proboscis; 2. elongated rostrum; 3. defined neck; 4. three digits on each foot. (*b*) Artistic reconstruction of macraucheniids (Mike Keesey). (Online version in colour.)

Located in the upper sector of the Raudal del Gauyabero panel, this zoomorphic image distinctly features an animal with anatomical elements characteristic of Litopterna. The head is considerably shorter dorsoventrally than anteroposteriorly, which gives the appearance of an elongated rostrum. Moreover, the rostral portion prominently displays a well-developed extension that is semi-uniform in thickness and resembles a proboscis. In comparison to this distinct head, the long neck is similar to that of camelids and the posterior of the animal is sizeable. Presented in a quadrupedal pose, the limbs appear semi-gracile and notably exhibit three digits on both the forefoot and hindfoot. Although extant South American tapir species are known to have proboscises, general characteristics like tetradactyl forelimbs [73] and a stout neck are fundamentally dissimilar to this painting from La Lindosa. Overall, the presented fauna displays morphological traits (i.e. proboscis, defined neck, long limbs and three digits on each foot) that we interpret to be reminiscent of the extinct South American litopterns (figure 7; see the electronic supplementary material for details of taxonomy, detailed morphological characteristics and habit).

Along with differences in anatomy, *Macrauchenia* and *Xenorhinotherium* appear to have distinctive geographical distributions. Cartelle & Lessa [74] suggest biogeographic segregation between *Macrauchenia patachonica* from Bolivia to southern Chile and *Xenorhinotherium bahiense* from Brazil & Venezuela [75]. In the Pampas region, the presence of *Macrauchenia* sp*.* bones is documented from Arroyo Seco 2, a locality with lithic artefacts and evidence of human hunting/scavenging of megafaunal material dated to approximately 14.1 ka [61]. At the Centinela del Mar locality, a date of 13.2 ka was obtained from *M. patachonica* specimens, further illustrating the potential contemporaneity with early humans in South America [76]. Although the evidence is not as abundant in the northwestern region, *X. bahiense* has been recorded from Taima-Taima in Venezuela [77,78], a fossiliferous locality that dates to approximately 15*–*16.1 ka. All in all, there is a likely spatio-temporal overlap between Litopterna and early human settlers in the northwestern region of the South American landscape, which supports the prospect of *Xenorhinotherium* being represented in the La Lindosa rock art.

## 5. Concluding remarks

Collectively, our understanding of La Lindosa human history and rock art has been transformed by the new dating of multiple rock shelters establishing the Late Pleistocene human colonization of the region, the discovery of a whole new section of the Serranía de la Lindosa with rock art, and the analysis of potential Ice Age megafauna depictions. In the absence of direct dating of the paintings our identification of the potential Ice Age megafauna is based on, (i) the naturalistic appearance and diagnostic morphological features of the animal images, (ii) the late Pleistocene archaeological dates from La Lindosa confirming the contemporaneity of humans and most of the megafauna depicted in rock art in the region, (iii) the recovery of ochre pigments in late Pleistocene archaeological strata, and (iv) the widespread depiction of extinct megafauna in rock art across the Americas. Ochre nodules are not direct evidence of the execution of Pleistocene-age red-painting megafauna designs, however, their presence in their earliest contexts along with the other lines of evidence presented in this paper, make it reasonable to suggest that they were used for painting.

Although we run the risk of having selected just a few features from the etic perspective of alien observers (e.g. [79]), we have shown that there are sound grounds to support the argument that paintings at La Lindosa likely represent now-extinct megafauna and the paintings are ancient. The timing of the peopling of the Americas [80] and the predatory relationship of humans to megafauna in South America is a hotly debated topic, where every line of evidence comes under high levels of scrutiny [58]. However, archaeological evidence clearly demonstrates that human entry into South America predated the extinction of megafauna by at least 1000 years [58], so it should come as no surprise that these early arrivals depicted the animals they encountered in their artwork. In addition, there is no doubt that the earliest artists from La Lindosa, which were among the earliest *Homo sapiens* to colonise Amazonia, were capable of conceptualizing and employing symbols and making and using art. Humans migrating to the New World had a rich tradition of image-making, including rock art paintings. Whether this tradition arrived with the first immigrants into the Americas, or whether this tradition developed independently in South America, is still unknown, although it seems most likely that art was part of the cultural repertoire of the first migrant groups.

The La Lindosa potential Ice Age rock art is not an anomaly and the available data demonstrate that rock painting was widespread during the LP-EH transition across the Americas. Unlike the Upper Palaeolithic art of Europe where there are multiple repetitions of rock art, the La Lindosa potential megafauna depictions are constituted by isolated finds in a small number of panels. This is similar to the picture from other regions of South America [15], Australia [3], South East Asia [2] and Africa [8]. The findings at La Lindosa contribute to the emerging picture of considerable geographical and stylistic variation in both geometric and figurative paintings that occur in rock shelters with early human occupations [13,81,82]. Some of the purported megafauna representations at La Lindosa are comparatively large, they are located in the middle or upper part of the panels with respect to today's ground surface, exhibit less cluttering of surrounding images, and are generally accompanied by an assemblage of animated human figures of diminutive size (figure 3). Detailed analysis of the juxtapositions and superimpositions in the parietal sectors where these paintings occur needs to be carried out, but we can propose as a working hypothesis that these large, ‘monumental' paintings were the first to be created followed by gradually smaller ones at lower heights on the panels. This temporal arrangement of paintings has similarities to the ‘Large Naturalistic Animal Period' of Australia [83] and observations made by Vialou & Viaolu [39] at Santa Elina, who found that large, uncluttered, naturalistic paintings are often located in the central and top sections of rock walls. More work and dating of the paintings will be required to test this hypothesis and to explore to what extent the variety of early art mimics the diversity of material culture across South America [84].

The La Lindosa early rock art probably played a role in identity formation and territoriality of the colonizing foragers of the northern Amazon. The areas initially occupied by the first human arrivals in South America were probably those with the highest ranking in food and/or resources [85]. Productive ecotones, such as La Lindosa, that exhibit forest-savannah-riverine mosaics with palm-dominated forests, would have been attractive for early foragers for the establishment of temporary or semi-permanent camps [86]. The late Pleistocene was a period of exploration of these empty spaces in which early pioneers constructed and defined their place in the landscape. The ‘appropriation' of privileged locales like La Lindosa, possibly took place through the marking of the landscape, creating images on the permanent, imposing rock walls that support the prominent table top ‘tepuis’. Future advances in the direct dating of rock art, along with the full exploration of La Lindosa, will allow us to better support or refute arguments about the extraordinary Ice Age rock art discussed in this paper. Dating of organic binders and silica skins are promising avenues to pursue next (e.g. [87–89]).The development of innovative dating programmes for these sandstone rock shelters will be crucial for determining the age of these paintings which will, in turn, improve our understanding of the early art and symbolism of the La Lindosa first settlers and the Americas as a whole.

## Data Availability

This article has no additional data.
